# Supporting the scholarship of teaching and learning: a department-based approach to faculty development

**DOI:** 10.1186/s12909-026-10800-y

**Published:** 2026-11-11

**Authors:** Agnes Elmberger, Terese Stenfors

**Affiliations:** Department of Learning, Informatics, Management and Ethics, Karolinska Institutet, Stockholm, SE-171 77 Sweden

**Keywords:** Faculty development, Professional development, Scholarship of Teaching and Learning, Health Professions Education, Situated learning, Collegial learning, Educational change, Co-design, Mixed methods

## Abstract

**Background:**

Faculty development is pivotal for supporting teachers in health professions education, yet concerns remain about its value in terms of change in practice. Attention is therefore shifting towards offering activities situated in practice and providing faculty with opportunities to develop their own local practices, focusing more on the Scholarship of Teaching and Learning as well as collegial interactions and teamwork. This study reports how a new faculty development programme focusing on scholarship and local change work was designed, and how the programme supported faculty in their Scholarship of Teaching and Learning and collegial networking.

**Methods:**

We describe the co-design and implementation of an eight-month, department-based faculty development programme at a medical university, built around scholarship and the development and documentation of a scholarly educational project. Drawing on both qualitative and quantitative data, we explore participants’ and mentors’ experiences of the programme.

**Results:**

Participants reported that the programme contributed to their professional growth and development as teachers and strengthened their capacity and mindset to pursue scholarly educational development. Working closely with other faculty members from the same department enhanced opportunities for feedback and collaborative learning, and the programme was perceived as creating a platform for continued networking beyond its duration.

**Conclusion:**

This study provides an example of how faculty development can be purposefully designed to address key concerns in the faculty development literature related to transfer and change in practice. The findings highlight the value of a longitudinal design and locally situated programme that enables collegial interaction and collaboration, and suggest that supporting faculty in developing as scholars strengthens their capacity to improve teaching and enact educational change in practice.

**Supplementary Information:**

The online version contains supplementary material available at https://doi.org/10.1186/s12909-026-10800-y.

## Introduction

Faculty development (FD) is key for supporting faculty in their continuous development as teachers and for improving student learning, and FD is now common in health professions education across the world [1, 2]. FD activities span from one-off workshops and lectures to longitudinal year-long programmes, focusing on teaching effectiveness and educational skills as well as leadership and professional development [1, 2]. While research has shown effect on the level of the individual teacher, such as change in skills and attitudes, research still struggles to show the value of FD in terms of change and development of teaching practices [1–4]. There is now more attention on moving beyond the previous focus on skills and knowledge in teaching and learning, towards providing situated FD activities that offer faculty opportunities to develop their own local teaching practices, with a heavier focus on change and scholarship [1, 2, 4, 5].

In this paper, we present how a new FD programme focusing on scholarship and local change work was designed and carried out, how participants perceived the programme, and the lessons we learned during the process. The overall aim of the study was to explore if and how the programme supported faculty in their Scholarship of Teaching and Learning (SoTL), and if it stimulated collegial networking and relationship building. Through this, we strive to offer useful insights that can inform future FD efforts aiming to support the development of SoTL.

## Conceptual framework

The programme is broadly grounded in sociocultural theories on learning, acknowledging interactions between people and context as essential. More specifically, learning is understood as a social and situated process, shaped through interaction with others and participation in the practices and activities of a community or workplace, rather than as the individual acquisition of knowledge [6]. These ideas have informed contemporary perspectives on FD, shifting attention from individual knowledge acquisition towards workplace-based initiatives embedded in practice, which understand learning as situated, social and constructed [4, 7]. Further, current approaches to FD often draw on principles of experiential learning [8], emphasising active engagement with authentic real-life problems, and the application of learning to participants’ own workplace contexts [7]. Aligning with this, the programme seeks to support participants in hands-on work and experiences while minimising time for didactic teaching.

More specifically, the conceptual framework for the programme is focused on three interconnected areas we identified in the FD literature: the need for situated FD, the growing focus on SoTL, and the importance of collegial interactions and communities of educators. Together, these areas reflect a sociocultural understanding of learning as emerging through active participation in practice and interaction with others.

### Situating faculty development in practice

Research shows that FD activities are often appreciated by the participating teachers who gain new educational knowledge and change their attitudes toward teaching and learning [1]. However, research also indicates a transfer problem where FD activities rarely lead to change in teaching practices and do not contribute to change at group or organizational levels [1–3, 9, 10]. This has partly been related to the fact that traditionally, many FD activities have been delivered to teachers in off-site settings such as in FD units, and that learnings from one context may not readily transfer into other contexts [1–4]. As a result, researchers and faculty developers emphasise a need for situated FD, sometimes referred to as the ‘situated turn in FD’ [2, 11]. Here, programmes and activities are situated in the settings in which teachers work and teach, their workplace, creating better opportunities for transfer and implementation [1, 2, 5, 7, 11]. In other words, instead of bringing teachers to off-site FD activities, it is argued that FD should move closer to the teaching practice [2, 4, 5, 7, 11]. By making FD a situated and adaptive practice, research suggests that teachers are enabled to develop and implement solutions that work in their own workplace contexts [2, 5].

### The scholarship of teaching and learning

The Scholarship of Teaching and Learning (SoTL) is receiving increasing attention and is partly linked to the professionalization of the teaching role seen within higher education over the last decades [12, 13]. The definitions of SoTL are many, but it can broadly be understood as a systematic and evidence-informed approach through which teachers reflect on, explore, and develop their teaching practice [13, 14]. It includes scholarly explorations of one's teaching with the aim of improving it, drawing on existing literature and contributing to the shared knowledge base by publicly sharing the results of the inquiry [10, 12, 13, 15]. SoTL thus differs from implementing educational changes solely within one’s own teaching practice, by also involving the systematic investigation of those changes, grounding them in existing scholarship and research, and sharing outcomes with others [11, 12, 14]. As such, SoTL emphasises inquiry, reflection, and dissemination, and positions teachers not only as practitioners, but also as scholars who generate and share knowledge about educational practice [13].

Lately, researchers and faculty developers have been arguing for a greater focus on SoTL within FD to support faculty in developing as scholars while they work with practical initiatives that develop and improve their teaching practices and the learning of their students [1, 2, 5, 16, 17]. This way, a focus on SoTL in FD could also help with overcoming the problem of transfer and change following FD. However, challenges for SoTL identified by faculty include competing priorities, lack of time and lack of knowledge of relevant literature and research methodologies, and previous research has shown that such challenges could be overcome by participating in structured FD initiatives focusing on SoTL [15].

### Collegial interactions and communities of educators

The third pillar in the conceptual framework of our programme design concerns the heavy focus on individual teachers in FD, which has been highlighted as another aspect connected to problems with transfer and implementation following FD [1, 4, 5, 11]. Often, FD activities focus on developing individual teachers’ knowledge and skills in teaching and learning. However, this creates difficulties in implementing change following FD activities as individual teachers often struggle to influence the practices of a larger group of individuals [3, 18] and as the workplace context often has considerable impact on faculty’s practices [4, 16, 19]. The focus on individual teachers also has negative consequences for collegial exchange and learning, further complicating the implementation and change of teaching practices in the workplace [5, 20]. To address these issues, it has been suggested for FD initiatives to focus on groups of teachers who normally work together [4, 5, 16, 20]. In line with this, we have seen a shift towards more collaborative FD initiatives, focusing on mentoring, peer support and the creation of professional networks of educators, where activities are designed to provide greater opportunity for interactions [4, 15, 21, 22]. As an example, Kim et al. (2021), found that support from peers in a FD programme was a key asset for participants in overcoming challenges that they had experienced doing SoTL research. Such emphasis on collegial interactions in which peers learn from and interact with each other is important for establishing communities of educators that can sustain beyond FD activities and support continuous change and development in teaching [21, 23].

In summary, to follow recommendations from previous research and to address current challenges in FD, we designed a programme that would focus on SoTL. As proposed in previous research, we focused the programme around a scholarly project to support participants in developing and applying skills and learning and engaging in scholarship [1, 5, 21]. Further, the scholarly projects were chosen by participants to ensure practical relevance to their own teaching practices. Second, the programme was locally situated within one department and held over the duration of eight months. Finally, the programme was designed around peer interaction and support to create opportunities for learning with and from colleagues and to enable participants to develop peer networks and communities of educators in their own workplace [1, 4, 21, 22]. In addition, mentors were recruited, also locally from the department, to further support collegial learning and interaction. In this way, the programme design incorporated situated learning, emphasising learning as participation in practice; SoTL, framing teachers as investigators of their own educational practice; and collegial learning, highlighting learning as a social process shaped by interaction with others. Together, this aligns with our sociocultural understanding of learning as situated, social and constructed, rather than as an individual acquisition of knowledge.

## Programme design and structure

### Setting

The setting for the programme and study was Karolinska Institutet, a single-faculty, research-intense medical university in Sweden, offering numerous programmes at master and bachelor levels, doctoral education, and professional education. Teaching at the university is research based, meaning that faculty often are involved in both research and teaching, and many also in clinical work. For certain teaching roles, such as programme director or course director, ten weeks of qualifying courses in higher education teaching is required.

The current programme was designed and delivered to one of the university’s departments which hosts three master programmes and numerous courses at all levels within, for example, health economics, medical education, health informatics, medical management, bioentrepreneurship and healthcare ethics. Faculty at the department have backgrounds in areas such as healthcare, psychology, economics, higher education, informatics, biology, and engineering, and whilst many of them are experienced teachers, some did not yet fulfil the formal criteria of ten weeks of training, hence the department management sponsored the development of this programme.

### Programme design

The overarching purpose of the programme was formulated as follows: “to stimulate the Scholarship of Teaching and Learning by supporting the participant in a scholarly project aimed at improving their own teaching practice”. Several intended learning outcomes were specified for the programme, in alignment with the intended learning outcomes for qualifying courses in higher education teaching as specified by The Association of Swedish Higher Education Institutions [24]. The design was established with the Unit for Teaching and Learning at the university to verify that the programme was in line with the university’s overarching vision for FD and to ensure that the programme would be easily transferable to other departments in the future.

We adopted a co-design approach in the planning phase of the programme and interviews were performed with four participants and two mentors, before the programme started, to inform the design of the programme activities and to explore participants’ needs. These interviews, lasting between 17–29 min, indicated that teachers at our department wanted space and time for discussing and reflecting on teaching and learning together with other teachers and wanted to connect with more people at the department. They also expressed that they lacked protected time for developing their teaching and pursuing ideas for improvement, that they wanted to get inspired to do new things, and that they had ideas and plans for working scholarly in relation to their teaching but lacked support and knowledge to do so.

### Overview of the curriculum

The programme took as a starting point a pre-existing online, self-paced, course provided at Stockholm University [25], offered to individual faculty and focusing on documenting a scholarly educational project. The online course consisted of eight modules, with the primary objective of enabling participants to develop, implement, and evaluate their own scholarly educational project. Participants individually submitted their text-based assignments through the online platform, culminating in a final written report of their projects.

The online modules were supplemented with five half-day, on-site seminars at the department spread out over eight months. Each seminar had a specific focus relating to the online modules, e.g. idea generation, research methodology, and implementation and change management, and included preparatory work such as reading and/or preparing a presentation of the project progress. These seminars were intended to provide participants with opportunities for group reflection and discussion, and each seminar included time for discussing and receiving feedback on participants’ work from peers, mentors and programme facilitators. An online chat was created in Teams, aiming to provide further opportunity for questions, discussions and reflections between the on-site seminars. Literature and other resources were also provided through the Teams chat.

Furthermore, aligning with our conceptual framework, teachers were recruited at the department and paced to take the online course together as a cohort, rather than individually. Also, each participant was paired with an individual mentor from the department to support the participants in developing their project. Instructions to mentors included that they would attend all on-site seminars, offer support and feedback between seminars corresponding to a total of 10 h (it was up to the participant and mentor to monitor and decide the form of this, but it could include meetings, reading of text materials, e-mailing etc.), and read and feedback on all text assignments including the final report (this was monitored by programme facilitators). In case the mentor was absent from an on-site seminar, a separate meeting was to be arranged with the mentee in addition to the 10 h of supervision between seminars.

In summary, these additions aimed to facilitate collaborative learning, foster the creation of local educational networks at the workplace, and enhance opportunities for practical implementation.

### Assessment and credits

Completion of the full programme corresponded to four weeks of full-time study; three weeks for completion of online modules and one week for active seminar participation. There was also an option to undertake a less extensive version of the online modules, yielding two weeks full-time study. The online modules and the final report were assessed by examiners at Stockholm University.

## Programme participants and projects

The programme was advertised via e-mail to the whole department, inviting participants with over five weeks of prior FD participation, but less than 10, and mentors with over 10 weeks of prior FD participation. Some mentors were also approached with individual e-mails by the programme facilitators. Seven participants and seven mentors signed up for the programme. Participants and mentors were paired by programme facilitators to ensure that they did not have a professional relationship such as manager/employee and where possible, shared interests or methodological expertise.

Five of seven participants finished the programme and passed the final examination. Their projects are described in Table 1. One participant dropped out after the first on-site session, stating lack of time to pursue the programme as the reason. One participant completed most assignments but did not hand in the final examination, again stating lack of time as the reason.

**Table 1:** Summary of projects developed in the programme

A qualitative exploration of students’ and supervisors’ experiences of workplace-based education
Developing and implementing a new teaching method
A qualitative exploration of students’ perceptions and understandings of the subject area
Building and evaluating a new teaching case
A mixed methods evaluation of a study programme

## Material and methods

The study is based on a social constructivist approach, acknowledging individuals’ various perspectives on reality and that participants and researchers together co-construct meaning [26]. The research was undertaken using primarily a qualitative approach but with supporting survey data, focusing on exploring participants’ and mentors’ experiences of the programme. Data was collected after the programme had finished using an exit survey and interviews. The study was vetted by the Swedish Ethical Review Authority (no: 023–05303-01).

## Participants, data collection and analysis

### Interviews

All participants and mentors were invited to participate in individual interviews after the programme had finished. They were invited by researcher AE by e-mail and reminded once if no reply had been received. This resulted in a total of nine interviewees; four programme participants and five mentors, their demographics are summarized in Table 2.

**Table 2:** Interview participant characteristics

		**P​articipants**	**Mentors**
Gender			
	Male	0	1
	Female	4	4
Age (years)			
	Range	40–50	45–65
Teaching experience in higher education (years)			
	Range	6–20	7–25
	Mean	12,5	19,8
PhD			
	Yes	4	4
	No	0	1

The interviews were semi-structured and followed an interview guide developed for this study, with questions covering areas such as feasibility of the programme activities, experiences of the programme, perceived changes in knowledge, attitudes, and competence including development as scholar, and collaborative learning and networking [see additional file 1]. Mentors were interviewed using a similar guide, but with fewer questions about the development of the educational projects.

All interviews were performed by a research assistant who had not been involved in the programme. They were performed using online conference software, voice-recorded and lasted between 30–54 min (mean 40 min). Informed consent was obtained from all participants before interviews. The interviews were transcribed verbatim by the research assistant and researcher AE and TS only had access to the anonymized transcripts.

Transcripts were analysed using reflexive thematic analysis [27, 28]. Both researchers read all transcripts to familiarize themselves with the data and initiated the analysis by making notes and highlighting relevant passages in the transcripts. Thereafter, researcher AE coded all transcripts, sorted codes into groups which were then sorted into sub-themes and themes through an iterative process moving between the data and emerging interpretations. Throughout the analysis, discussions were held between the researchers to reflect on, discuss and review the interpretations, explore alternative understandings, and refine the themes being generated. Finally, transcripts were read through again to search for any uncoded data and to ensure themes provided a coherent and just interpretation of the data, before the final results were agreed upon and quotes were chosen and translated into English.

### Survey

A survey was e-mailed to all programme participants after the programme had finished. The mentors received no survey. The survey was developed for this study and included both standardised programme evaluation items provided by the university, as well as items added for the purpose of this study [see additional file 2]. The items were grouped into three categories: general programme evaluation, collaborative learning and networking, and perceived effects. We asked respondents to rate each item using a five-point Likert-style scale according to how well they agreed with the statement: 1 = fully disagree, 2 = partly disagree, 3 = neutral, 4 = partly agree, and 5 = fully agree. The survey also included three open-ended items.

The survey was distributed using the web-based platform REDCap electronic data capture tool hosted by Karolinska Institutet [29]. All six invited participants finished the survey, and all included items, yielding a response rate and an item response rate of 100%. The items were analysed descriptively and reported as frequencies. The responses to the open-ended questions were added to the qualitative data and analysed using the reflexive thematic analysis described above.

### Reflexivity

Both researchers were engaged in the design and facilitation of the programme and we acknowledge the potential impact our involvement in the programme might have on analysis and results. For this reason, interviews were conducted by a research assistant not involved in the programme and we continuously discussed and reviewed our perspectives during the analysis of data. We also made efforts to inform participants that the aim of our study was not about evaluating them as participants in the programme, but to study the programme with the overarching aim of improving it for future iterations. Our preliminary results were discussed with faculty at the university during a teacher congress to strengthen confirmability, and with faculty from other institutions nationally and internationally at a conference to strengthen transferability. Both researchers have worked in FD and conducted research in this field for a number of years.

## Findings

**Fig. 1:**
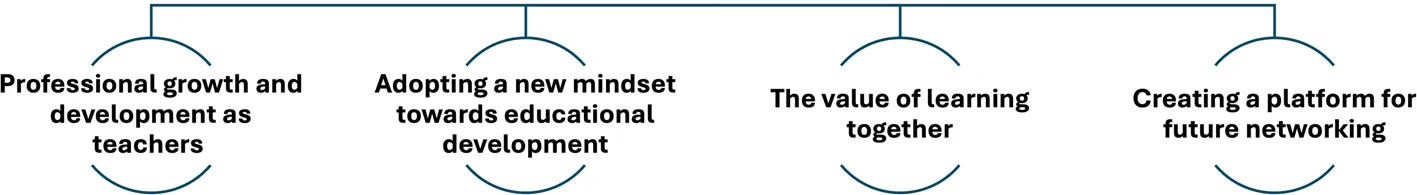
Four themes that describe how participants perceived the programme in relation to their scholarly development and collegial learning

**Fig. 2:**
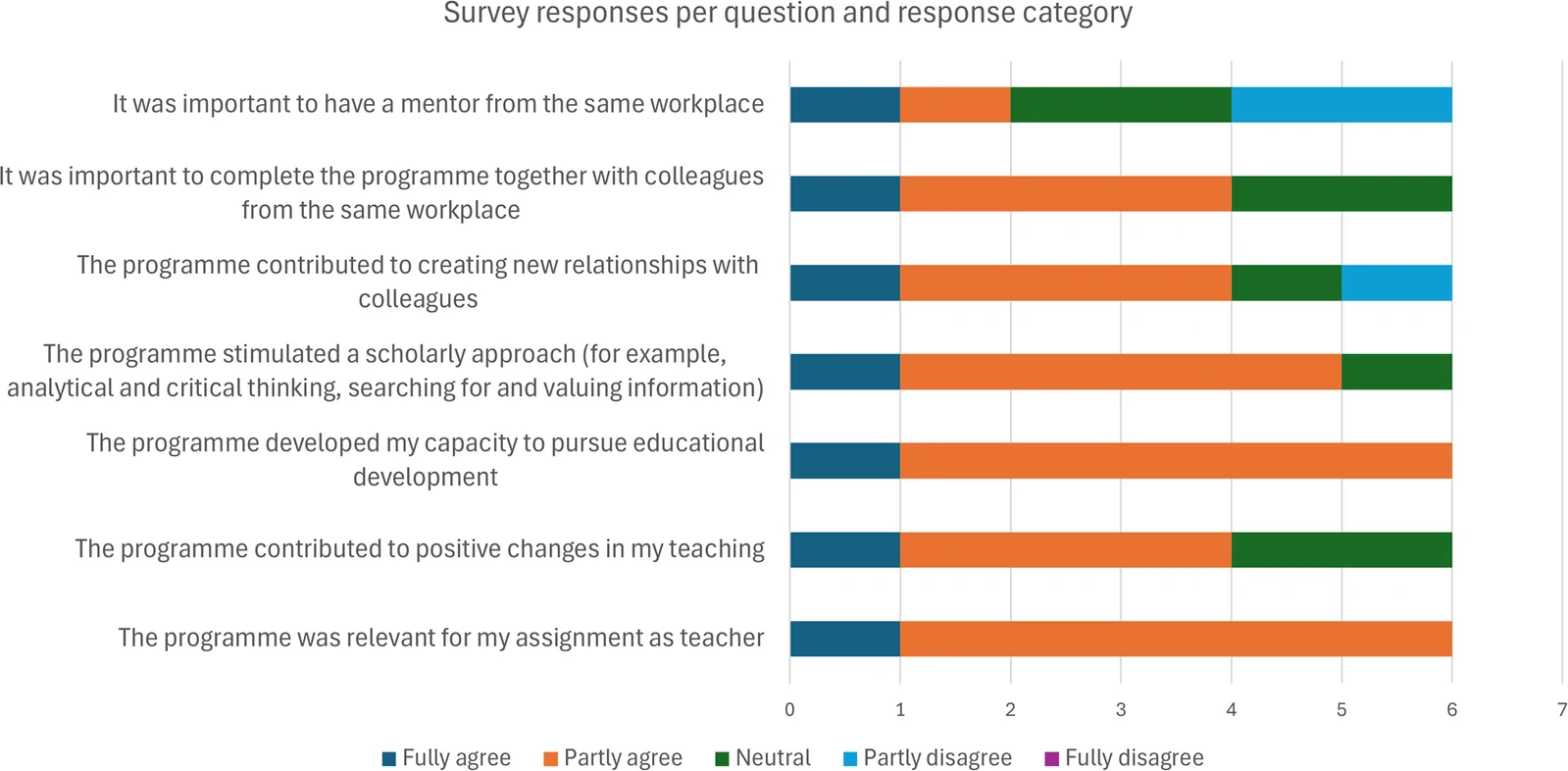
Summary of survey responses from programme participants

Analysing the interviews and the survey data, we arrived at four themes that describe how participants perceived the programme in relation to their scholarly development and collegial learning (Fig. 1). Quotes from interviews with participants are designated with P and mentors with M. Open-ended text responses from the survey are designated with the record ID number. To preserve anonymity, personal information has been removed from quotes. Summary of survey responses can be found in Fig. 2.

### Professional growth and development as teacher

All participants partly (5/6) or fully agreed (1/6) that the programme was relevant for their teaching assignment. In the interviews, participants emphasized that they grew in their role as teachers throughout the programme. Some participants, who were more junior in their teaching trajectories, identified that they had acquired both theoretical knowledge and skills within teaching and learning, such as gaining insights into new teaching methods and concepts. For others, who could be described as more senior both in terms of teaching experience as well as previous FD participation, the programme did not bring any major new insights into teaching methods or educational theories. Rather, they, together with the more junior participants, described having developed in their teaching role, such as feeling more secure in their roles as teachers, adopting a more reflective approach towards their teaching, and the programme adding to their “overarching educational compass” (P3). Another participant suggested:

*“Well, if we sum it up, I’m more secure in my role, so it’s already had an impact, and it will be permanent I think”* (P1)

In the survey, all participants (6/6) fully agreed that the programme had contributed to their development as teachers, with one stating in the open-ended follow up question:

*“The course has contributed to my development as a teacher in several ways, from inspiring literature with new concepts and models to valuable discussions with colleagues.” (open-ended response, ID 4)*

Participants also described how the programme had led to improvements in their teaching. In the survey, 4/6 participants partly or fully agreed that the programme had resulted in positive changes in their teaching, while 2/6 were neutral. In the interviews, several participants raised that an important aspect of the programme was that they themselves were free to choose what to focus on in their projects, meaning that the projects were relevant and directly applicable in their own teaching practice. Further, participants described that the programme had contributed to other developments in their teaching practice, not directly related to their projects. For example, one participant described getting valuable insights into supervision and mentoring from being in a mentee position during the programme, creating a greater understanding for students and their learning processes, and felt they had developed into a better supervisor. Another participant described how the programme had contributed to development of their teaching and that they now had decided to reduce the number of courses they were teaching in because of the programme, as they had realised there is more to teaching than content knowledge:

*“I decided to reduce the number of courses that I will be involved (in), because I realized that it’s not enough […] just having subject knowledge […] so then I decided to reduce the number of courses, to be able to focus more on this development part.”* (P2)

Mentors also described that the programme added to their learning and professional development, and that they learned through discussing and working with their mentees and following their work with their projects. As one mentor described it:

*“My role nowadays is much about leadership, being a manager, […] I don’t have much time to do research myself, but being a mentor is a way for me […] it’s professional development for myself, and I enjoy that a lot, and the one I was mentoring was working with a project that’s relevant for my teaching and the work I lead.”* (M1)

### Adopting a new mindset towards educational development

In addition to developing in their teaching roles, all participants suggested the programme had developed their capacity to pursue educational development (partly agreed: 5/6 and fully agreed: 1/6). In the interviews, participants highlighted how they adopted a new mindset towards educational development throughout the programme. At the start, some felt unfamiliar with the underlying ideas in the programme about scholarship and educational development projects. They were unsure what an educational development project should entail and had difficulties visualizing their final products at the end of the programme.

*"What exactly is an educational development project? Is this it? I was very unsure whether my project actually qualified as one or if it was more leaning towards a research project, and then I also didn't know if it was enough to be able to say that I’m developing something educational […] like, what is the end product?"* (P1)

While many had experiences from research within other fields such as clinical or basic science research, some grappled with education as a research field and were not acquainted with educational or social science research from before. Connected to this, some found it difficult searching and finding relevant educational research, not being accustomed to such literature from before. In terms of coming from other backgrounds, some were also new to qualitative research methods, and while some had chosen qualitative methods for their projects, they expressed doubting whether qualitative explorations of their teaching would be of interest to others. However, they also described this as one of the major learning moments in the programme, and that they after finishing the programme felt more able to apply qualitative methods to research their own teaching practice in the future.

During the development of their projects, and throughout the programme, participants described adopting a different approach towards educational development and scholarship. Many were working with course development from before, trying new things, implementing new teaching methods, and developing their courses in various ways. Yet, they now described that the programme had enabled them to move towards a more systematized process, seeing a difference between what they referred to as “standard” course development, and a more scholarly, investigating approach, researching their practice and connecting the development to other research and literature. This was reflected also in the survey, where most participants partly (4/6) or fully agreed (1/6) that the programme had stimulated a scholarly approach.

*"The last part of the programme really pushed for not just doing a pedagogical development project, but actually turning it into a research study. I truly see a huge opportunity for several of the teachers in the department who teach so much that they hardly do any research at all (...) to then take a programme like this, be pushed, and be given the time and space to conduct their own little research study."* (P3)

Mentors also described seeing a difference between course development and scholarly work more clearly after the programme, and described moving towards a more scholarly approach towards their own educational practice and development:

*"We work a lot on continuously developing our teaching (...) pedagogically speaking, we’re always working on it, but we rarely do it as scholars, so to speak, we just do it within our little group, but we never pass it on (...) and I found it very rewarding to see the potential in what we do, like that it can also be a project that you can present to colleagues (...) with the intention of sharing it, and not just ‘this is my little thing that I do to develop my course,’ but rather thinking more like, ‘okay, if I read up a bit more broadly’, if I think ‘how could it be useful to more people, how can I share it?’" (M2)*

In summary, both participating teachers and mentors described having moved towards a more scholarly approach in their educational development throughout the programme, and participants also reflected on the continuation of this process:

*“This (the programme) was a step on a journey that I hope will never end, you know (...) my own goal was this project I was working on, to give it time and space and to discuss it with others so that it could be published, and it's well on its way.” (*P3)

### The value of learning together

Participants as well as mentors noted great value in the on-site seminars of the programme, adding opportunity and space for feedback, reflections, and learning together. They described how the on-site seminars enabled them to discuss and deliberate their ideas with others, or “to hash things out together” as one participant put it. The fact that participants and mentors had different backgrounds and previous experiences further added to valuable conversations, getting another set of eyes and perspective on the projects they were working on, leading to new ideas and contributing to building new knowledge together. Participants further described that the discussions within the group added motivation and joy to their work with the projects as well as to their work as teachers.

*"We had a session where we had read articles that we discussed in group at the on-site seminar. I thought that was really good. Then, of course, it was also great to get feedback and discuss each other’s projects, so I actually found all the on-site meetings very good and rewarding, and I would have liked to have even more of them." (P1).*

Connected to the collaborative and collegial aspects of the on-site seminars were the dynamics and relationships within the group. Some participants had anticipated that as they were all colleagues, potential previous interpersonal disagreements may have been brought to the classroom or that power imbalances and problems such as insecurity or feeling pressure to perform in front of each other could occur. However, several grew to realize that this was less of a problem than anticipated, and rather described learning from each other:

*"Everyone has thought about it on beforehand, maybe read an article or something. And it’s always so cool, even though we work at the same department, it’s still amazing to hear what others have noticed that I hadn’t seen. Like, ‘wow, you’re right’, and how we then collectively create more knowledge together by enriching what I’ve observed with what others have seen—that’s really awesome. So, it was fun." (P3)*

Many talked about the rather small group size as a positive aspect of the programme, as well as the informal discussions and relationship building that was going on during coffee breaks at the on-site seminars. This was also raised in the open-ended survey questions, where one noted:

*The seminars were good, and the coffee breaks made it enjoyable, which is not an insignificant factor in a smaller group where you want to create a sense of belonging to facilitate collaboration and openness*. (Open-ended response, ID 2)

### Creating a platform for future networking

Finally, although participants knew each other from before to various degrees, mainly having insights into each other’s research or work, participants suggested that the programme had given them the opportunity to get to know each other from an educational point of view and on a more personal level. It opened doors to others’ teaching, giving participants better insight into what others were teaching and working with and thus providing a basis for educational discussions.

*"I think it’s super valuable in that it opens doors to other colleagues’ teaching and makes it easier to collaborate, as I get insight and an entry point. Cause research, you often know what they’re researching, but you don’t know how they teach, how much, or what methods they use (…) Getting to know colleagues from a teaching perspective makes it easier to ask things or say, 'can you, or could you come in and give a lecture on this?*'" (M2)

This was reflected in the survey, with 4/6 participants agreeing that the programme had contributed to creating new relationships with colleagues. The fact that the programme was built around a group of people from the same department was described as an added value and an important feature of the design. It enabled participants to build new relationships over time, where people developed a sense of connection with colleagues whom they otherwise did not work with. It also enabled them to build relationships across research groups and departmental divisions. In this way, the programme contributed to creating a context and platform from where to network and build new collaborations. For example, one participant described that the programme had helped in enabling a new collaboration, having invited a participant to give a lecture in their course, and another one said:

*“I’ve been working at the department for quite a long time now and I know all colleagues, but working from a pedagogical, or reflecting from a pedagogical perspective, I would say that maybe that (the programme) gave a platform for me to initiate future networking.”* (M3)

In the survey, participants rated the importance of completing the programme together with colleagues from the same workplace higher (4/6 partly or fully agreed), compared to the importance that mentors were from the same workplace (2/6 partly or fully agreed). In the open-ended responses, one participant stated:

*“Supervisors do not need to be from the same department. The selection of supervisors then becomes too limited. I would have preferred a supervisor with greater educational expertise. My supervisor was kind but did not provide much guidance on, for example, how to frame and narrow down the project so that it became relevant to my educational context, rather than something general and less useful.” (Open-ended response, ID 5)*

In summary, participants and mentors valued that the programme focused on collaboration and felt that the programme had enabled them to build new relationships that could potentially lead to future collaborations.

## Discussion

In this paper, we have presented the development of a new FD programme focused on SoTL and participants' own scholarly projects, locally situated in the workplace and designed around collaborative learning and peer interaction among colleagues of teachers. We explored if and how the programme supported faculty in their SoTL and collegial networking, with results showing several important aspects for future FD which we discuss below.

First, we focused the programme on SoTL and the findings suggest that the design supported the participants in a more scholarly investigation of their practices. As identified in previous research [15], and the interviews we performed prior to the programme, faculty have difficulty finding time, space and support to work with SoTL, and lack knowledge about educational research. In this programme, faculty overcame that through the structured design focused on projects. They described having developed a new approach and mindset towards scholarship, understanding it as an investigative approach going beyond curriculum development. Rather than merely acquiring research skills, participants appeared to begin positioning themselves as scholars by engaging in inquiry into their own teaching and through interaction with colleagues. This is in line with our intentions focusing on SoTL and suggests that the programme helped participants gain new knowledge, experience and confidence in scholarship. In addition, some faculty with significant teaching duties lack space and time for disciplinary research [15, 30] and thus an initiative like this where their work can be framed as academic practice might be especially important. We also believe that the focus on reflection, sharing one's process and getting feedback from others during the on-site seminars was important for their development as scholars, creating space for experimentation and sharing of challenges, an important part of SoTL, which has also been discussed in previous research [30]. Further, the programme was designed to offer participants experience of scholarly work around their teaching, exposing them to educational research and relevant research skills, and participants described having developed their capacity as scholars.

Second, we designed the programme to foster collegial interactions and network building by inviting faculty and mentors from a single department. The findings suggest that peers learned from each other during the programme and participants described that these interactions had stimulated the development of new relationships among them and offered opportunities for future collaborations. This supports previous research favouring a focus on colleagues rather than individuals in FD [5, 21, 30], and that such a focus may better contribute to organisational and collective change [16]. It also resonates with recent work on communities of practice in faculty development, highlighting the importance of sustained interaction, participant engagement, and opportunities for shared reflection in supporting learning [31]. Together, this aligns well with sociocultural perspectives which understand learning as supported by interactions with others [6]. From this perspective, the collegial interactions described by participants can be understood as an important mechanism for learning through which participants exchanged experiences, understandings of teaching and developed new approaches to their practice. Further, research by Roxå and colleagues [32] shows that what they call ‘significant conversations’, i.e. conversations where teachers share experiences and practices and which are important for learning and meaningful change to happen, often takes place backstage such as during coffee breaks, just as described by the teachers in our study. This is further explored in other research, again showing that proximity plays an important role for significant conversations to take place [33]. In addition, significant conversations can be encouraged by creating a conducive space for discussions between faculty from different disciplines [33], and while participants in our programme worked at the same department, they held different disciplinary backgrounds and described exchanging ideas and experiences leading to greater learning. However, for significant conversations to take place, individuals must have trustful relationships [33], which may be a potential problem when faculty come from the same workplace. They might be less willing to take the risk to engage in vulnerable conversations about uncertainty, complexity and maybe even failure in teaching, compromising the readiness to challenge one's assumptions about teaching and learning, and thus significant conversations may not appear. This is in line with other research, emphasizing that it is essential to have safe environments characterized by mutual trust, feeling safe enough to voice insecurities or challenges, experiment and innovate [30]. We thus believe there must be continuous work on how to support these conversations and fostering such a safe space, and while the programme design around collegial interaction appeared to contribute to collaboration and networking, this design may warrant more active work around trust and reflection about interpersonal relationships throughout the course of the programme. One way to do so, which was also voiced by participants, was having enough time for informal discussions during coffee breaks to build relationships as well as spending most of the time during on-site seminars for discussion and reflection within the group.

Third, and in relation to the previous point, delivering the programme on-site aimed to situate the learning in the context in which faculty work to create greater opportunities for transfer and implementation. In comparison to traditional FD, where participants are participating in off-site activities, focusing on general teaching and learning with subsequent challenges for transfer and change in practice [1, 2, 4, 11], this programme had several added values. We designed it around scholarly projects, and as participants were free to choose what to focus on, it offered an opportunity to develop a project that would be directly applicable and relevant to their teaching. This was especially highlighted as positive by participants, and by working with projects in their own workplace setting, participants described being able to develop knowledge and practices within the same context in which they would later be applied. While this study did not focus specifically on the implementation of participants’ projects, they described that the focus on projects had enabled change and implementation in their teaching practices, and the findings thus suggest that the programme may have offered a way through which participants engaged in change of their teaching practices. This supports other research stressing the need to move beyond delivering theoretical knowledge in off-site activities, to allowing participants to work with change in a locally situated programme [1], again aligning with the understanding that learning is inseparable from context [6, 7].

In terms of strengths and limitations of the study, we acknowledge that the results build on only one iteration of the programme and thus a small sample of participants. We are also aware that our involvement as programme facilitators might influence the participants’ willingness to comment negatively on the programme. For this reason, interviews were performed by a research assistant not involved in the programme. We recognize that most participants were women, however this reflects the teacher composition at our department with a majority of women engaged in teaching and educational development. Further, we aimed to include both the description of the process behind the development of the programme, as well as the study of the programme effects, to ensure continuous development of not only our programme, but also to generate insights that others may benefit from when building and developing their own FD programmes. Finally, the data builds on participants experiences and we did not assess actual change or implementation following the programme, which would be an important next step in further research.

In summary, this study demonstrates how FD can be purposefully designed to address persistent concerns in the FD literature regarding transfer and change in practice. Participants described that the department-based, longitudinal programme design, with its focus on scholarly inquiry and collegial learning, supported them in growing as teachers, scholars and members of an educational community. Also, the co-designed approach helped us design the programme in line with participant needs. Taken together, our findings suggest that FD may better support change in teaching practice when it is designed as a longitudinal, locally situated, and collective endeavour that integrates scholarly projects with structured opportunities for collegial interaction, rather than focusing primarily on individual skills acquisition. This study contributes a model for such FD that may be useful for faculty developers and educational leaders. Key design aspects include a scholarly approach focusing on educational projects, chosen by participants, anchoring their work in educational theory and research, and offering protected space for networking and relationship building by situating the work in the context of participants. Future research could explore how such programmes influence longer-term educational practice and change.

## Supplementary Information

Additional file 1: Semi-structured interview guide.

Additional file 2: Survey questionnaire. Survey questionnaire, distributed using the web-based platform REDCap electronic data capture tool hosted by Karolinska Institutet.

## Data Availability

The datasets generated and/or analysed during the current study are not publicly available due to risk of compromising participant anonymity, given the small sample size and the contextual nature of the qualitative data.

## Abbreviations

FD: Faculty development
SoTL: Scholarship of Teaching and Learning
