# Supplementary material for: Supporting the scholarship of teaching and learning: a department-based approach to faculty development
Source: BMC Med Educ. 2026 Nov 11;26:1845. doi: 10.1186/s12909-026-10800-y (PMC13757019; doi:10.1186/s12909-026-10800-y)
Supplement: Supplementary file 1 [file 12909_2026_10800_MOESM1_ESM.docx]

**Introduction**

- Presentation of the project’s purpose and pedagogical development:
  - Partly course evaluation
  - Partly research, aiming to examine teachers’ educational development and whether a course like this is useful and can contribute to such development
- Inform that participation in the interview is **voluntary**, that the participant may withdraw at any time without giving a reason, and that everything said will remain confidential *(we take several measures to minimize the risk that data can be linked to individual participants: all data is stored on a secure server, all data is coded, interview quotes are anonymized, and any identifying information in the interview is replaced with pseudonyms).*
- Informed consent form are provided *(these have also been sent to the participant in advance).*
- Any questions?

**Section 1: Background and Programme Experience**

1. Why did you decide to take this programme?

2. Can you describe your overall experience of completing the programme?

- What aspects did you find particularly rewarding or valuable?
- Were there any parts that felt challenging, difficult, or unclear?
- To what extent do you feel the programme met your needs for pedagogical development?

**Section 2: Learning and Development**

3. What have you learned during the programme?

- Can you give specific examples of new knowledge or skills?
- If you feel you did not learn anything new, can you explain why?

4. How did you experience working on the pedagogical project within the programme?

- Was this your first time doing this type of project?
- What worked well, and what was more challenging?

5. How, if at all, has the programme influenced your ability to carry out similar pedagogical projects?

- Do you feel more confident now?
- Do you plan to continue working on similar projects in the future?

6. Has the programme influenced your own teaching practice? If so, how?

7. Has the programme affected how you view your role as a teacher? If so, in what way?

8. Do you think the programme will have an impact on your future career?
*(For example: teaching qualifications/credits, applying for assistant professor status, publications, or other opportunities?)*

**Section 3: Collegial Learning and Networks**

9. How did you experience the opportunities to network and discuss with colleagues during the programme?
*(Optional follow-up: Were these opportunities sufficient? Why or why not?)*

10. What was it like taking the programme together with colleagues from the same workplace?

- What were the advantages?
- Were there any disadvantages?

11. How was your experience of being supervised by a colleague from the same workplace?

- What were the advantages?
- Were there any disadvantages?

12. Do you feel that the programme contributed to creating new networks or professional relationships?

- If yes, how?
- If not, why do you think that was?

13. Did the programme lead to pedagogical discussions with colleagues outside of the formal programme setting?

- Can you give examples?

**Section 4: Closing Questions**

14. Do you have any suggestions for how the programme could be improved?

- What changes would you recommend, and why?

15. Is there anything else you would like to add that we haven’t covered?

16. Do you have any questions for me?

Thank you very much for your time and valuable insights.
