# Supplementary material for: Supporting the scholarship of teaching and learning: a department-based approach to faculty development
Source: BMC Med Educ. 2026 Nov 11;26:1845. doi: 10.1186/s12909-026-10800-y (PMC13757019; doi:10.1186/s12909-026-10800-y)
Supplement: Supplementary file 2 [file 12909_2026_10800_MOESM2_ESM.docx]

SURVEY

**Educational Scholarship and Project Work: An Advanced Faculty Development Course**

This survey concerns the programme *Educational Scholarship and Project Work: An Advanced Faculty Development Course,* which you have recently completed.

Your responses to this survey will help us further develop the programme and will also be used for evaluating the programme and included in a research paper. Your participation is voluntary, and all responses will be anonymous.

Many thanks for your participation!

Please indicate your level of agreement with the following statements:

| Statement | Fully agree | Partly agree | Neutral | Partly disagree | Fully disagree |
| --- | --- | --- | --- | --- | --- |
| The programme was relevant for my assignment as teacher. | ☐ | ☐ | ☐ | ☐ | ☐ |
| The programme contributed to positive changes in my teaching. | ☐ | ☐ | ☐ | ☐ | ☐ |
| The programme developed my capacity to pursue educational development. | ☐ | ☐ | ☐ | ☐ | ☐ |
| The programme stimulated a scholarly approach (for example, analytical and critical thinking, searching for and valuing information). | ☐ | ☐ | ☐ | ☐ | ☐ |
| The programme contributed to creating new relationships with colleagues. | ☐ | ☐ | ☐ | ☐ | ☐ |
| It was important to complete the programme together with colleagues from the same workplace. | ☐ | ☐ | ☐ | ☐ | ☐ |
| It was important to have a mentor from the same workplace. | ☐ | ☐ | ☐ | ☐ | ☐ |

**Open-ended Questions**

1. Has the course contributed to your development as a teacher? (Yes / No)

If yes, please describe how the course has contributed to your development as a teacher:

If no, please explain why the course has not contributed to your development as a teacher:

__________________________________________

2. Were there any parts of the course that were excellent? (Yes / No)

If yes, please specify which parts of the course were excellent and in what way:

__________________________________________
3. Do you have any suggestions for how the course could be improved? (Yes / No)

If yes, please specify what could be improved and how:

__________________________________________
